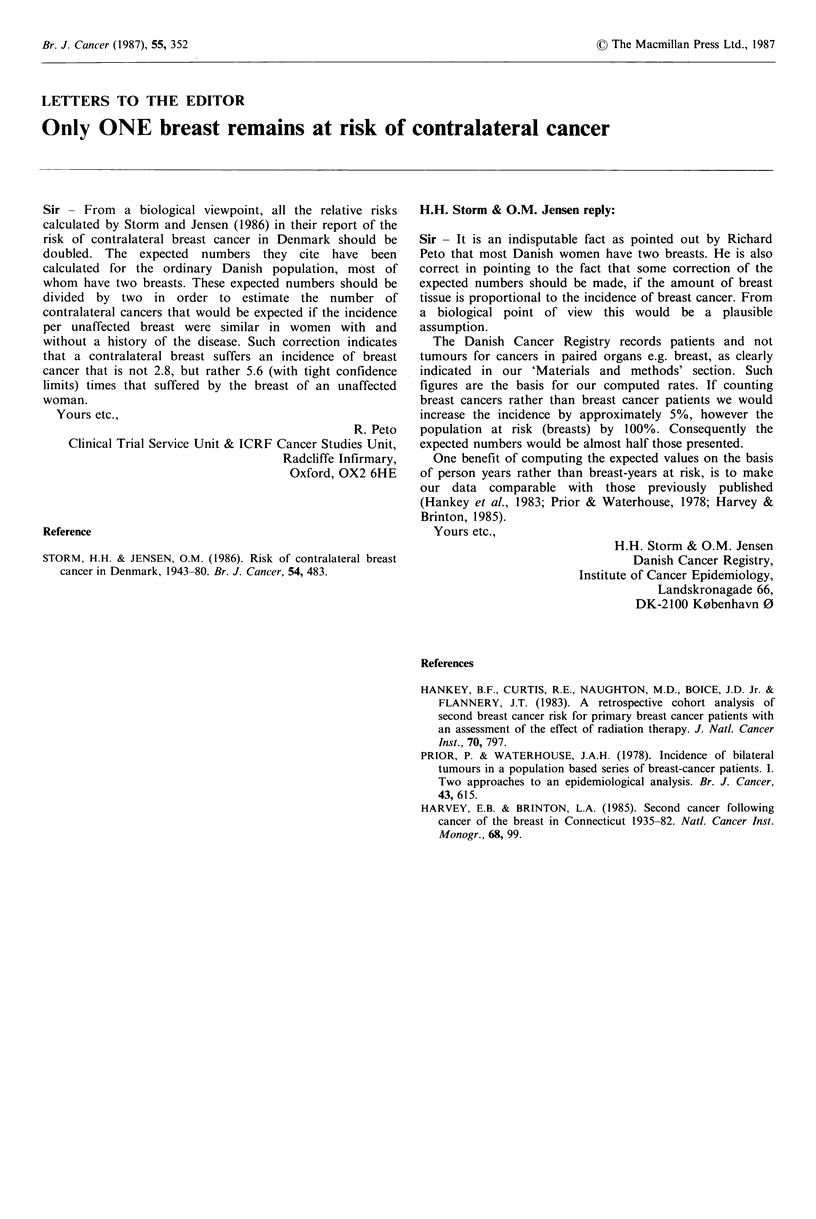# H.H. Storm & O.M. Jensen reply

**Published:** 1987-03

**Authors:** H.H. Storm, O.M. Jensen


Yours etc.,

R. Peto
Clinical Trial Service Unit & ICRF Cancer Studies Unit,
Radcliffe Infirmary,
Oxford, OX2 6HE

**Reference**

STORM, H.H. & JENSEN, O.M. (1986). Risk of contralateral breast cancer in Denmark, 1943–80. *Br. J. Cancer*, **54**, 483.

**H.H. Storm & O.M. Jensen reply:**

**Sir** – It is an indisputable fact as pointed out by Richard Peto that most Danish women have two breasts. He is also correct in pointing to the fact that some correction of the expected numbers should be made, if the amount of breast tissue is proportional to the incidence of breast cancer. From a biological point of view this would be a plausible assumption.

The Danish Cancer Registry records patients and not tumours for cancers in paired organs e.g. breast, as clearly indicated in our 'Materials and methods' section. Such figures are the basis for our computed rates. If counting breast cancers rather than breast cancer patients we would increase the incidence by approximately 5%, however the population at risk (breasts) by 100%. Consequently the expected numbers would be almost half those presented.

One benefit of computing the expected values on the basis of person years rather than breast-years at risk, is to make our data comparable with those previously published (Hankey *et al.*, 1983; Prior & Waterhouse, 1978; Harvey & Brinton, 1985).

Yours etc.,

H.H. Storm & O.M. Jensen
Danish Cancer Registry,
Institute of Cancer Epidemiology,
Landskronagade 66,
DK-2100 København Ø

**References**

HANKEY, B.F., CURTIS, R.E., NAUGHTON, M.D., BOICE, J.D. Jr. & FLANNERY, J.T. (1983). A retrospective cohort analysis of second breast cancer risk for primary breast cancer patients with an assessment of the effect of radiation therapy. *J. Natl. Cancer Inst.*, **70**, 797.

PRIOR, P. & WATERHOUSE, J.A.H. (1978). Incidence of bilateral tumours in a population based series of breast-cancer patients. I. Two approaches to an epidemiological analysis. *Br. J. Cancer*, **43**, 615.

HARVEY, E.B. & BRINTON, L.A. (1985). Second cancer following cancer of the breast in Connecticut 1935–82. *Natl. Cancer Inst. Monogr.*, **68**, 99.